# A one-week 5-choice serial reaction time task to measure impulsivity and attention in adult and adolescent mice

**DOI:** 10.1038/srep42519

**Published:** 2017-02-15

**Authors:** Esther Remmelink, Uyen Chau, August B. Smit, Matthijs Verhage, Maarten Loos

**Affiliations:** 1 Sylics (Synaptologics B.V.), 1008 BA Amsterdam, The Netherlands; 2 Department of Molecular and Cellular Neurobiology, Center for Neurogenomics and Cognitive Research, Neuroscience Campus Amsterdam, VU University, 1081 HV Amsterdam, The Netherlands; 3 Department of Functional Genomics, Center for Neurogenomics and Cognitive Research, Neuroscience Campus Amsterdam, VU University, 1081 HV Amsterdam, The Netherlands

## Abstract

Many psychiatric disorders emerge during adolescence. The study of executive functions in animal models of these disorders critically requires short-duration tasks measuring these functions before the animal ages. Here, a novel 5-choice serial reaction time task (5-CSRTT) protocol is presented, to measure attention and impulsivity within one week, without scheduled food deprivation and with little animal handling. Mice were allowed 24-h/day task access from their home-cage, during which they could self-pace task progression and earn unlimited food rewards depending on task performance. Manipulation of task parameters in this self-paced 5-CSRTT protocol (SP-5C) affected attentional performance and impulsivity to a similar extent as previously observed in the 5-CSRTT. Task activity followed intrinsic circadian rhythm, distinctive for the SP-5C protocol, with task performance stable over the day. The sensitivity of the SP-5C protocol to detect strain differences between C57BL/6J, DBA/2 J, BXD16 and BXD62 mice was demonstrated as well as its suitability for testing adolescent mice. Acute administration of the muscarinic acetylcholine receptor antagonist scopolamine impaired attentional performance, providing initial pharmacological validation of the task. The SP-5C substantially shortens the assessment of impulsivity and attention, increases test efficiency and enables the assessment of adolescent mouse models of psychiatric disorders.

Executive functions, such as attention and inhibitory control, are affected in many psychiatric disorders, e.g. schizophrenia^1^, attention-deficit hyperactivity disorder (ADHD)^2^ and substance abuse disorders^3^. Most of these psychiatric disorders emerge during adolescence^4^ and are likely due to the major development during this period of the prefrontal cortex, which controls executive functions^4^,^5^. Rodent models are instrumental in understanding normal development and dysregulation of executive functions, but operant tasks used to assess these functions require prolonged training periods, precluding their use during the adolescence time-window. For example, the five-choice serial reaction time task (5-CSRTT) for rats^6^,^7^ and mice^8^, in which the animals have to respond to a brief stimulus presented pseudorandomly in one of five holes in order to obtain a food reward and which measures aspects of visuospatial sustained and divided attention^9^, as well as a form of impulsive action called ‘waiting impulsivity’^10^,^11^, typically takes months to complete. Adolescence, however, spans only a few weeks in rodents^12^,^13^, limiting the applicability of this widely-used operant task to adult subjects.

Besides prolonged training, other less desirable factors of operant testing protocols for rodents have been recognized. Most importantly, food deprivation, which is applied to motivate animals to perform the task, can affect impulsive behavior^14^. Additionally, animal handling causes anxiety and stress in mice^15^ and even after repeated handling heart rate and corticosterone levels may remain increased^16^,^17^. Adolescence has been proposed to be a period during which decision making capacity is more vulnerable to disruption by stressors^12^, and therefore it is desirable to test adolescent animals in an environment where stressors are limited. Furthermore, the combination of prolonged training, food restriction regimes, and animal handling renders the 5-CSRTT labor-intensive.

Considering these limitations of the 5-CSRTT, we set out to design a novel 5-CSRTT protocol that could sensitively measure attention and impulsivity during adolescence, without scheduled food deprivation and with little animal handling. In this protocol, mice were allowed 24-h/day continuous access to the task, during which they could task-dependently earn unlimited food rewards and could self-pace task progression. To achieve continuous task access and little human interference, we connected a home-cage to a commercially available operant 5-CSRTT chamber (named CombiCage hereafter). This self-paced 5-CSRTT protocol (SP-5C) was designed based on the ‘conventional’ 5-CSRTT protocol^18^, with automatic, criterion based, transition between training stages. However, in the conventional protocol mice were placed in an operant chamber for 25 min/day to perform the 5-CSRTT and were food restricted to decrease body weight to 90% of their initial weight before and during the task. To investigate the validity of the SP-5C protocol in the CombiCage as a procedure measuring attention and impulsivity, we extracted multiple performance measures equivalent to those obtained in the conventional 5-CSRTT^6^,^19^,^20^.

Mid-adolescent as well as adult C57BL/6J mice were submitted to the SP-5C to determine whether they could be trained to respond to a 1 s stimulus within a shorter period of time compared to the conventional protocol. Task parameters were varied to alter the attentional load and to prompt impulsive responding in the SP-5C. We compared performance of these two groups with the performance of C57BL/6J mice previously tested in the conventional protocol^20^,^21^.

Next, we investigated whether the 24-h/day continuous protocol was compatible with acute drug testing, an important application of the 5-CSRTT. Because the cholinergic system is known to play a crucial role in attention in humans as well as in rodents^22^, we assessed whether attention in the SP-5C was reduced by scopolamine administration, a muscarinic acetylcholine receptor antagonist, as was previously shown in mice in a conventional protocol^23^,^24^,^25^.

Lastly, to determine whether the SP-5C in the CombiCage delivers the sensitivity to detect strain differences in attention and impulsivity, as reported previously^19^,^20^,^21^,^26^, performance of C57BL/6J, DBA/2J, BXD16 and BXD62 in the SP-5C was compared.

We found that allowing mice continuous access to the 5-CSRTT greatly reduced experiment time. This made it possible to study attention and impulsivity during adolescence, without reducing the sensitivity to detect strain differences and drug effects in adult mice.

## Results

### Adolescent and adult C57BL/6J mice reached the performance criterion in the SP-5C within 1 week

**Figure 1:**
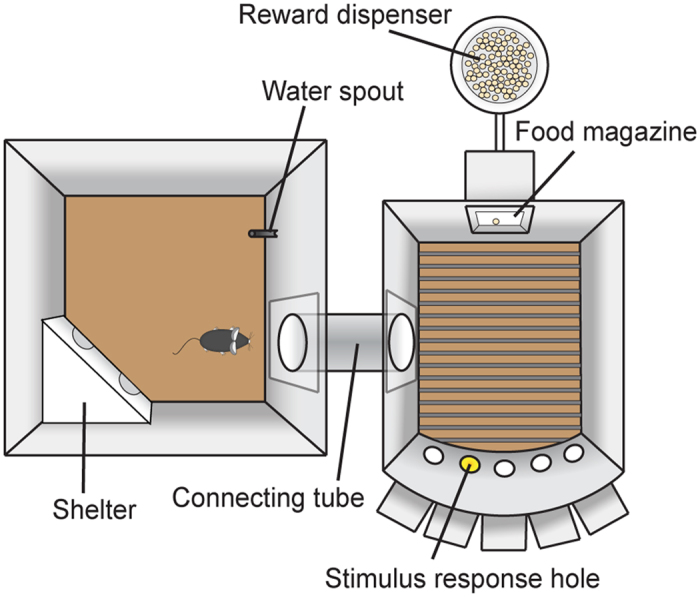
Schematic overview of the CombiCage. The CombiCage consisted of a home-cage (30 × 30 cm; left side) connected to a MedAssociates operant chamber (right side). The home-cage was equipped with a shelter and a water dispenser and became connected to the operant chamber via a connection tube (diameter 6 cm) just before the start of the protocol. The left wall of the operant chamber was modified to accommodate the insertion of this connection tube. The operant chamber was equipped with five nose poke units that could be illuminated with yellow cue LEDs and which contained infrared response detectors (stimulus response holes). On the opposite side of the chamber a food magazine was located connected to a pellet reward dispenser.

A group of adolescent and adult C57BL/6J mice, 5 and 10 weeks old respectively, were tested in the SP-5C protocol in the CombiCage (Fig. 1) where they had continuous access to an operant chamber from their home-cage and could participate in the task for 24-h/day. Progression through the training stages of the task was automated, based on criterion achievement, until the mice were able to respond to a 1 s stimulus. The only source of food was the reward pellets the mice could earn by performing the task, without a restriction on the number. Their performance was compared to adult C57BL/6J mice that were previously tested in the conventional set-up for 25 min/day^20^,^21^ and that were kept on a food restriction regime to decrease body weight to 90% of their initial body weight, according to 5-CSRTT standard procedures.

**Figure 2:**
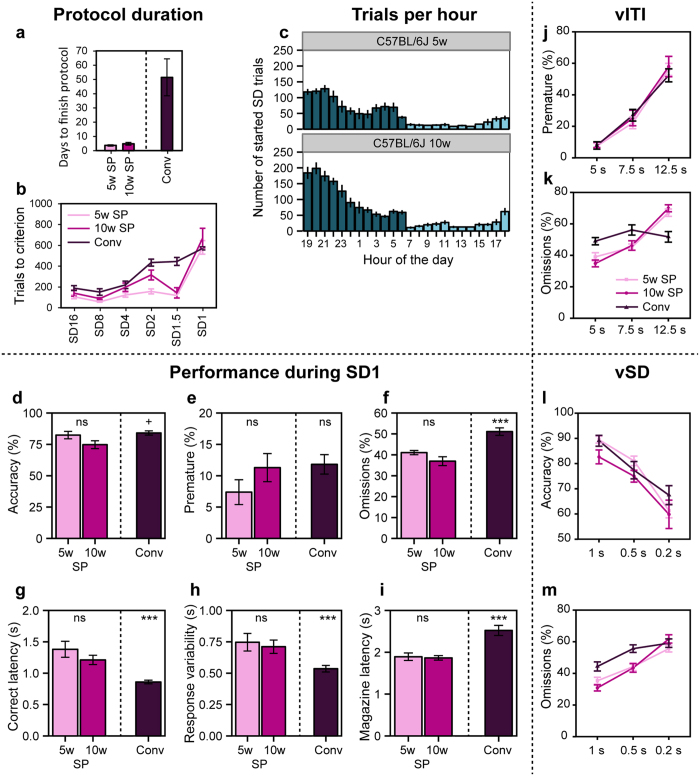
Performance of adolescent and adult C57BL/6J mice in the SP-5C compared to performance of adult C57BL/6J mice in the conventional 5-CSRTT. (**a**) Days to finish the protocol until 5 times criterion achievement in SD1 in the SP-5C (SP), or 10 sessions of SD1 in the conventional 5-CSRTT (Conv). (**b**) Number of trials to criterion (see methods) per SD stage for five and ten week old C57BL/6J mice in the SP-5C compared to adult C57BL/6J mice in the conventional 5-CSRTT. (**c**) Number of started SD trials in the SP-5C per bin of one hour. The dark phase started at 19 h and ended at 7 h. Dark blue bars represent hours of the dark phase, light blue bars the hours of the light phase. (**d–i**) Performance during SD1 compared between five and ten week old C57BL/6J mice in the SP-5C, and compared to performance during SD1 session 5–10 in adult C57BL/6J mice in the conventional 5-CSRTT. ^+^p < 0.10; ***p < 0.001; ns = non-significant. (**j,k**) Percentage premature responses and percentage omissions during the variable ITI stage. (**l,m**) Percentage accuracy and percentage omissions during the variable SD stage.

The mice in the CombiCage needed on average 4.3 days to finish the SP-5C protocol (Fig. 2a; Table 1), from magazine training to the completion of five stages with a stimulus duration of 1 s (SD1). Each stage was completed when a mouse reached the following performance criteria: started trials >50, accuracy >60% and [omissions <30% or number of correct responses > = 200]. The mice needed an additional 3.4 days to complete 500 trials in a variable ITI stage (vITI; fixed random ITI of 5, 7.5 and 12.5 s), 500 trials in a variable SD stage (vSD; fixed random SD of 1, 0.5 and 0.2 s) and 500 SD1 trials in between. In contrast, mice in the conventional 5-CSRTT needed on average 51.5 days to finish the protocol until 10 sessions in SD1. Hence, the novel task protocol was completed ~10 times faster.

Comparing the number of trials to criterion per SD stage in the SP-5C and conventional 5-CSRTT showed that in the SP-5C mice needed fewer trials to finish the protocol (Table 1), where the difference mainly arose from the different number of trials in the SD2 and SD1.5 stage (Fig. 2b). Nonetheless, the number of trials in SD1 was comparable (Fig. 2b).

The mice had access to the operant chamber for 24-h/day and could self-pace their task activity. Plotting the number of started trials over de hours of the dark and light phase of the day showed that the mice adjusted their task activity to their natural activity patterns as they started most trials of the SD stages during the dark phase (Fig. 2c). C57BL/6J mice started 83% of all SD trials during the dark phase Therefore, and because the contrast between the stimulus lights and the light in the environment was less during the light phase, we decided to hereafter only analyze and report performance measures based on trials started during the dark phase.

During SD1, there was a trend towards lower accuracy in the SP-5C compared to the conventional 5-CSRTT (Fig. 2d) (t(42) = −1.97, p = 0.055). The percentage of premature responses was not different in the two protocols (Fig. 2e) (t(42) = −1.07, p = 0.290). Omissions and magazine latency were lower in the SP-5C compared to the conventional 5-CSRTT (Fig. 2f and i) (respectively, t(42) = −4.80, p < 0.001 and t(42) = −4.43, p < 0.001), whereas correct latency and response variability were higher in the SP-5C (Fig. 2g and h) (respectively, t(42) = 6.61, p < 0.001 and t(42) = 4.09, p < 0.001). Five and ten week old C57BL/6J mice tested in the SP-5C were not significantly different on any of the performance measures (Fig. 2d–i; Accuracy: t(42) = −1.61, p = 0.114; Premature: t(42) = 1.10, p = 0.278; Omissions: t(42) = −1.03, p = 0.308; Correct latency: t(42) = −1.62, p = 0.112; Response variability: t(42) = −0.48, p = 0.634; Magazine latency: t(42) = −0.11, p = 0.917).

Accuracy and premature responses were influenced in the SP-5C during a variable ITI and a variable SD stage in a similar way as observed previously in the conventional 5-CSRTT. A main effect of ITI duration on premature responses was observed (χ^2^(2) = 126.52, p < 0.0001). Similarly, a main effect of SD on accuracy was detected (χ^2^(2) = 43.03, p < 0.001). No significant effect of protocol (CombiCage versus Conventional) on premature responses during the vITI stage was identified (t(41) = 1.41, p = 0.167), or on accuracy (t(41) = −1.68, p = 0.100) during the vSD stage. However, a significant interaction effect of stage and protocol on the number of omissions during the vITI stage was observed (χ^2^(4) = 30.58, p < 0.001). Omissions increased during this stage in the SP-5C but remained relatively constant in the conventional 5-CSRTT. A trend towards an interaction effect of protocol on omissions during the vSD stage was detected (χ^2^(4) = 9.34, p = 0.053). No effect of age on performance in the SP-5C during the vITI or vSD stages was perceived (vITI premature: t(41) = 0.14, p = 0.886; vITI omissions: t(41) = −0.65, p = 0.518; vSD accuracy: t(41) = −0.93, p = 0.355; vSD omissions: t(41) = −0.79, p = 0.432).

Over the 8 days in the CombiCage, adolescent C57BL/6J slightly gained weight, i.e. 1%, whereas adult mice lost 7%. They consumed 2.5 g and 2.6 g of reward pellets per day, respectively. Detailed information on food, weight, days and trials to finish is shown in Table 1.

Overall, these results show that adult as well as adolescent mice can be more efficiently tested in the SP-5C compared to the conventional 5-CSRTT protocol. Despite some differences between the two protocols in the absolute values of parameters, the novel protocol did not compromise the expected effects of manipulations of the ITI or SD on measures of attention and impulsivity.

### Scopolamine affects attention in the CombiCage

**Figure 3:**
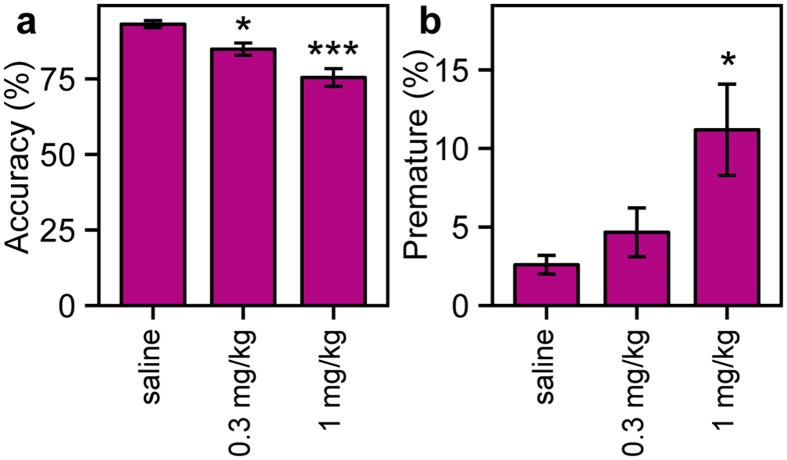
Effect of scopolamine on accuracy and premature responses in the SP-5C. (**a**) Scopolamine reduced accuracy in a dose dependent manner. (**b**) A trend towards an increase in premature responses due to scopolamine administration was detected. Post-hoc testing showed a significant effect on premature responses at 1 mg/kg scopolamine. *Dunnett post-hoc p < 0.05 ***Dunnett post-hoc p < 0.001.

In order to establish whether a 24-h/day protocol was compatible with acute drug testing and whether drug effects were comparable to those previously observed in conventional 5-CSRTT, we tested the effect of scopolamine, a mAChR antagonist known to reduce attention^23^,^24^,^25^, in C57BL/6J mice. Scopolamine reduced accuracy in a dose dependent manner in the SP-5C (Fig. 3a) (F(2, 43) = 8.40, p < 0.001) during the first 2 hours after drug administration. Both the 0.3 mg/kg and the 1 mg/kg dose significantly reduced accuracy compared to saline (t(43) = −2.84, p = 0.013; t(43) = −5.87, p < 0.001, respectively). A trend towards an increase in premature responses due to scopolamine administration was observed (Fig. 3b)(F(2, 43) = 2.94, p = 0.063). Post-hoc testing showed a significant effect on premature responses at only 1 mg/kg scopolamine compared to saline (t(43) = 3.26, p = 0.004). Scopolamine administration had no significant overall effect on the percentage of omissions (F(2, 43) = 1.32, p = 0.278), response variability (F(2, 43) = 1.51, p = 0.233), the number of started trials (F(2, 43) = 1.79, p = 0.180), magazine latency (F(2, 43) = 2.29, p = 0.114) or correct response latency (F(2, 43) = 0.29, p = 0.747).

Scopolamine was administered according to a Latin square design, in which mice were their own controls and received each dose of the drug. The day of administration had no effect on accuracy (F(2, 43) = 0.30, p = 0.741) nor on premature responses (F(2, 43) = 0.20, p = 0.822). Additionally, accuracy returned to pre-administration levels on the 2 days in between drug administration days (F(2, 51) = 1.69, p = 0.194).

These results demonstrate that the SP-5C protocol can be used for high throughput drug screening.

### Strain differences in attention and impulsivity can be reproduced in the SP-5C

**Figure 4:**
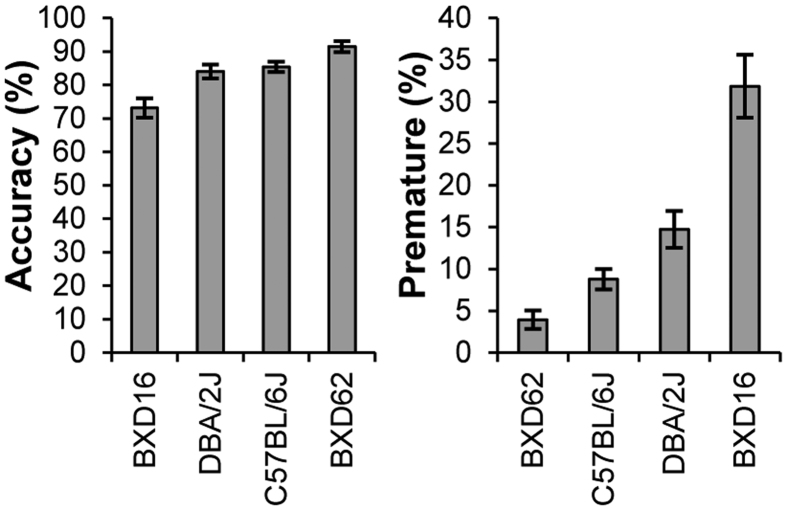
BXD strain performance in the conventional 5-CSRTT. Accuracy and premature responses in 4 BXD strains (C57BL/6J, DBA/2J, BXD16 and BXD62) during sessions 5–10 in SD1. BXD16 and BXD62 showed most extreme performance in the BXD panel previously tested. Data from Loos *et al*.^20^,^21^.

Of a BXD recombinant inbred panel that was tested previously in the conventional 5-CSRTT^20^,^21^, BXD16 and BXD62 mice showed most extreme attention and impulsivity during session 5–10 in SD1, as measured by the percentage accuracy and percentage of premature responses (Fig. 4). BXD16 mice showed low accuracy and high premature response levels, whereas BXD62 mice showed high accuracy and low premature response levels. Accuracy and premature responses of C57BL/6J and DBA/2 J mice were in between these two BXD strains (Fig. 4).

**Figure 5:**
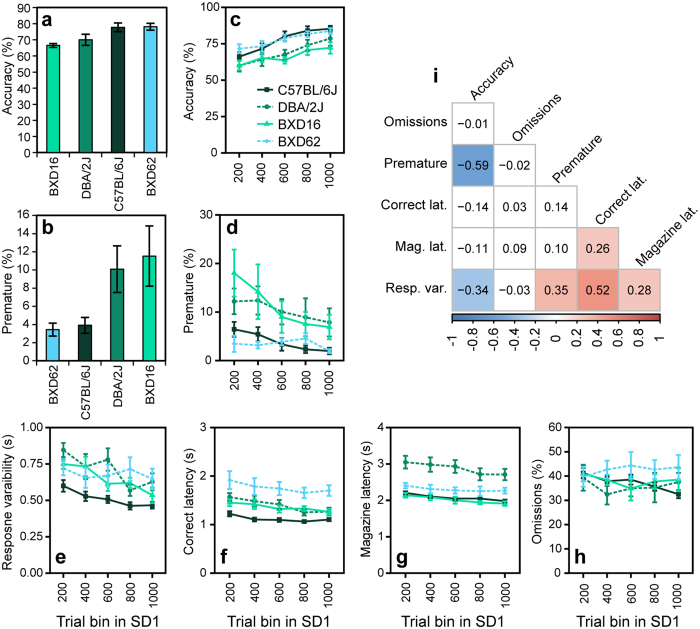
Performance of C57BL/6J, DBA/2 J, BXD16 and BXD62 mice during SD1 in the SP-5C. (**a,b)** Accuracy and premature responses during SD1 dark phase trials. **(c–h)** Performance during SD1, split into five bins of 200 trials (Trial 1–200, 200–400, 400–600, 600–800, 800–1000) before performance of dark phase trials was extracted. C57BL/6J n = 21, DBA/2J n = 10, BXD16 n = 6 and BXD62 n = 6. **(i)** Correlation matrix for the parameters as measured in the four strains during SD1. The numbers in the blocks indicate the Kendall tau correlation coefficient. Colored blocks represent significant FDR correct correlations with p < 0.05. Resp. var. = response variability, Mag. lat. = Magazine latency.

We assessed the performance of these four strains in the SP-5C to determine the sensitivity of this protocol to detect strain differences in attention and impulsivity. We observed strain differences in accuracy and premature responses during SD1 (Fig. 5a,b) (Accuracy: F(3, 39) = 2.92, p = 0.046; Premature: F(3, 39) = 5.16, p = 0.004). As expected, accuracy was lowest in BXD16 mice and highest in BXD62 mice, and for premature responses the opposite effect was detected, with BXD16 mice being most impulsive and BXD62 mice least. However, due to the small number of animals (n = 6 for these 2 strains) and large variation observed in BXD16 mice, these differences during all dark phase trials in SD1 did not reach significance (Accuracy: t(39) = 1.91, p = 0.236; Premature: t(39) = −2.29, p = 0.115). However, BXD16 mice did show significantly more premature responses than the 21 C57BL/6J mice (t(39) = 2.97, p = 0.025). Accuracy in DBA/2 J was not significantly different from accuracy in C57BL/6J mice (t(39) = −2.03, p = 0.191) but DBA/2 J were more impulsive than C57BL/6J mice (t(39) = 3.04, p = 0.020).

Data of the conventional 5-CSRTT shown in Fig. 4 was based on the last five sessions in SD1. To investigate how performance developed during SD1 in the SP-5C, we split the 1000 SD1 trials in bins of 200 trials after which only dark phase trials in these bins were used for calculating performance measures (Fig. 5c–h). These plots illustrated a more pronounced difference in accuracy between BXD16 and BXD62 mice at the end of SD1 (trial 600–1000) (Fig. 5c), whereas the difference in premature responses was more pronounced at the beginning of SD1 (trial 1–400) (Fig. 5d). Response variability, often considered a measure of lapses in attention^27^,^28^, did not show the same strain differences as accuracy (Fig. 5e). BXD16 and BXD62 mice showed overlapping values (z = 0.47, p = 0.964), whereas DBA/2 J mice showed higher variability than C57BL/6J mice (z = 4.46, p < 0.001). Strikingly, BXD62 mice showed a higher correct response latency than C57BL/6J mice (Fig. 5f) (z = 3.08, p = 0.011), whereas their accuracy levels were similar (z = 1.94, p = 0.208). Magazine latency, considered negatively related to motivation, was higher in DBA/2 J mice than in any of the other three strains (DBA – B6: z = 7.78, p < 0.001; DBA – BXD16: z = 6.24, p < 0.001; DBA – BXD62: z = 4.17, p < 0.001). Correspondingly, DBA/2 J mice earned fewer rewards per day (t(39) = 3.50, p = 0.006) and lost more weight during the experiment (Table 2). This different behavior of DBA/2 J mice was not due to general learning impairments, as these mice were not different from the other strains in the number of trials to criterion in stages SD16 to SD1.5 (DBA–B6: p = 0.102; DBA - BXD16: p = 0.912; DBA - BXD62: p = 0.468). However, a trend in DBA/2 J mice towards more started trials during all SD stages was observed (t(39) = 2.65, p = 0.053) (Table 2).

Omissions were not significantly different between strains (χ^2^(3) = 3.25, p = 0.354). They also were stable over bins (χ^2^(3) = 2.80, p = 0.100), whereas all other parameters only stabilized at the end of SD1 (comparing trial bin 600–800 to trial bin 800–1000: Accuracy: z = 1.43, p = 0.795; Premature: z = −1.57, p = 0.706; Response variability: z = −0.98, p = 0.967; Correct latency: z = −0.098, p = 1.000; Magazine latency: z = −0.78, p = 0.991). A significant interaction between strain and bin for response variability (χ^2^(12) = 24.09, p = 0.020) indicated that only this parameter stabilized differently in different strains.

Overall, the novel self-paced protocol can be used to discover genetic effects on performance by using different mouse strains, while providing additional information on how performance develops over time.

We correlated all performance measures during SD1 of all strains in order to determine whether constructs like attention, impulsivity and motivation can be measured independently in the SP-5C. A moderate but significant FDR corrected correlation (p < 0.001) was detected between premature responses and accuracy in this data set, indicating that the behavioral constructs attention and impulsivity were difficult to measure separately (Fig. 5i). Response variability, a parameter assumed to measure lapses in attention, was significantly correlated to accuracy (p = 0.003) as well as to premature responses (p = 0.002), magazine latency (p = 0.020), a measure of motivation, and to correct latency (p < 0.001), indicating this measure does not solely represent attentional processes. Because correct latency was correlated to magazine latency (p < 0.001), although weakly, it seems likely that this measure is partly a reflection of motivational processes in the SP-5C.

### Self-pacing of trials results in relatively constant task performance and motivation

**Figure 6:**
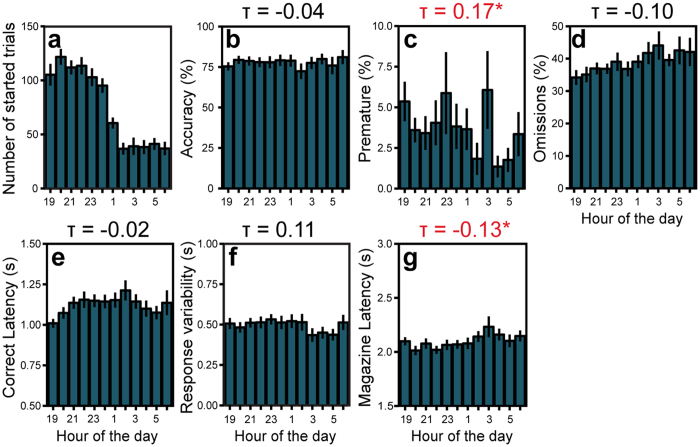
SD1 performance per hour of the dark phase. (**a**) Number of started SD1 trials of C57BL/6J mice per hour of the dark phase. (**b–g**) Average C57BL/6J SD1 performance shown for several measures per hour of the dark phase. The value above the bar plot represents the Kendall tau correlation coefficient between the performance measure per hour of the dark phase and the number of started trials per hour of the dark phase (correlation of fig. a with figs. (**b–g**) Red* numbers represent significant (p < 0.05) FDR corrected correlations.

The continuous self-paced start of trials in the SP-5C allowed us to study the effect of intrinsic circadian activity patterns (i.e. number of started trials) on task performance and motivation. The number of trials started per hour of C57BL/6J mice (Table 2) showed a strong circadian trend (Fig. 6a) similar to observed in Fig. 2c, with most trials started at the beginning of the dark phase. In contrast, task measures related to attention and impulsivity (Fig. 6b–f), as well as magazine latency (Fig. 6g), showed considerable less pronounced circadian rhythm during the dark phase. This suggests that self-pacing of trials has little effect on performance as it stays relatively constant over the day.

To quantify the relation between the number of trials started and task performance, we correlated the number of trials started in each hour of the dark phase with performance parameters calculated per hour. For this correlation analysis we used hourly data points per individual C57BL/6J mouse tested as part of the BXD panel, hence 252 (21 mice × 12 hours) data points per parameter. In general, correlations between the number of trials started and measures of performance and motivation were weak (Fig. 6b–g). Nevertheless, magazine latency was significantly negatively correlated to the number of trials started per hour, confirming the idea that mice started more trials when they were more motivated (i.e. lower magazine latency) to earn food. Interestingly, premature responses were significantly, but weakly positively correlated to the number of trials started per hour, indicating that impulsive behavior in the SP-5C is slightly influenced by activity. The other parameters, i.e. accuracy, omissions correct latency and response variability did not correlate with the number of started trials per hour, confirming that these parameters can be measured relatively stable over the day.

## Discussion

The 5-choice serial reaction time task (5-CSRTT) is the most widely used operant task to study attention and impulsivity in rodents. To overcome the limitations of prolonged training protocols, food deprivation and extensive and labor intensive animal handling, we designed a protocol in which mice have 24-h access to an operant chamber from their home-cage (CombiCage (CC)). This self-paced 5-CSRTT protocol (SP-5C) significantly shortened training duration, avoided pronounced weight loss and handling stress, and made it possible to study attention and impulsivity in adolescent mice. This amended protocol did not diminish the sensitivity of the 5-CSRTT to detect the effect of task adjustments, pharmacological interventions and strain differences, making it suitable for assessing attention and impulsivity in mouse models of neurodevelopmental disorders.

In the SP-5C, mice were trained to respond successfully to a stimulus of 1 s within one week, which is considerably faster than in a conventional 5-CSRTT protocol. Although this is largely the consequence of the higher number of trials per day in the SP-5C protocol, also the number of trials required to reach the SD1 stage in the SP-5C protocol was lower compared to the conventional protocol. Although the described protocol achieves a significant reduction in experimental time, the more labor intensive conventional set-up comes with the advantage that multiple subjects can be trained in the same box on a given day. Hence, whether a CombiCage setup has a higher or lower screening efficiency compared with the conventional 5-CSRTT depends on the number of cages available, labor force, and number of subjects that need to be tested per day.

The CombiCage set-up is unique in allowing mice self-paced task participation over the full day instead of confining the task to a short period, i.e. ~30 min of the day in a conventional 5-CSRTT. The number of trials started per hour of the day showed that C57BL/6J mice started most trials at the beginning of the dark phase, fewer at the end, and only few during the light phase. This pattern is similar to previously reported locomotor activity patterns over the day in home-cages^29^,^30^. Similarly, home-cage food intake occurs in peaks at the beginning and end of the dark phase in C57BL/6J mice^31^, which explains why mice started more trials at the beginning of the dark phase, during their natural foraging period. This indicates that mice titrate their task engagement towards their caloric needs. Hence, the motivation to perform the task is maintained throughout the dark phase by adjusting the number of initiated trials. Magazine latencies, an index of motivation, did not show a clear circadian effect during the dark phase. Moreover, none of the attention and impulsivity measures appeared to show strong circadian effects as observed for the number of trials started. This revealed that attention and impulsivity can be measured relatively independent from variation in activity, and that performance of mice remained relatively stable throughout the dark.

Absolute performance values obtained during SD1 trials in the SP-5C protocol were not always similar to those previously obtained in the conventional 5-CSRTT. The average correct latency, a measure of decision making speed, was higher in the SP-5C than in the conventional 5-CSRTT, as well was response variability, a measure of lapses in attention^19^,^20^,^27^,^28^. In addition, a trend towards lower accuracy in the SP-5C was observed. However, it should be noted that by including a 20 s consumption interval in the SP-5C protocol we presumably affected the number of omissions by removing a source of omissions related to consumption.

Despite the differences in absolute values, the difference in training protocol did not compromise the expected effects of adjustments of task variables, i.e. making the ITI and SD variable, on measures of attention and impulsivity. Shortening the stimulus duration, aimed at increasing the attentional load of the task, decreased the percentage of correct responses as a function of SD. Increasing the ITI, aimed at provoking impulsive responses, increased the percentage of impulsive premature responses. The effects of these manipulations were perfectly comparable to the effects observed in the conventional 5-CSRTT, with overlapping accuracy and premature response values. Although, omissions did change in the SP-5C when the ITI increased, which was not detected previously in the conventional 5-CSRTT in our lab, others did observe a similar effect on omissions^8^,^32^.

Comparisons of the observed correlations between parameters in the SP-5C in the present study and correlations observed in the conventional 5-CSRTT previously^32^,^33^, further corroborate the idea that the SP-5C evaluates the same behavioral phenomena as the conventional 5-CSRTT. Premature responses and accuracy correlated negatively in the SP-5C, as described previously^32^,^33^. The absence of a correlation between omissions and accuracy implies that these two parameters measure different aspects of attention, which also has been reported previously^20^. Although some variation between strains existed in the degree of weight loss due to this food regime, weight loss averaged over all mice was 4% for adult mice while most adolescent mice slightly gained weight during the task. In contrast, most published food restriction regimes during operant training protocols maintain mice at 80–90% of their body weight. We previously showed that removing access to standard chow generated sufficient motivation for mice to learn the contingencies of food acquisition, while largely preserving bodyweight during a four day discrimination learning protocol^34^. Current results expand these findings and show that unrestricted, but task performance dependent food availability for a period up to 18 days does not affect body weight, and can be considered a refined and efficient procedure for home-cage based testing of attention and impulsivity.

The SP-5C protocol and the conventional protocol differ with respect to several putative stress-inducing factors. In the SP-5C protocol the experiment duration is decreased to weeks rather than months, the food regime leads to less stress-inducing weight loss^35^,^36^,^37^, and stress-inducing and possibly confounding animal handling^15^,^16^ is reduced. On the other hand, whereas in the conventional 5-CSRTT it is possible to group-house rodents in between each training session, in the current CombiCage design mice cannot be group-housed. Although the consequences of individual housing of mice is a matter of debate^38^,^39^, group-housing is generally assumed to be advantageous for mice. Nevertheless, overall the SP-5C in the CombiCage seems a less stressful method to assess cognitive performance in mice.

Adolescence is a period during which the prefrontal cortex increases its connectivity with other brain regions^4^,^5^. Functions such as attention and inhibitory control, dependent on the prefrontal cortex, might therefore still be developing. Multiple studies found adolescent mice and rats to be more impulsive in a delay discounting task^40^,^41^,^42^,^43^, however no large differences in accuracy or impulsivity emerged between adolescent and adult mice in the SP-5C. It is possible that impulsive action has already reached mature levels during adolescence whereas the ability to delay gratification it still developing. Others have proposed that the development in human decision making capacity from mid-adolescence onwards is limited, but that decision making processes might be more vulnerable to disruption by stressors during adolescence^12^. The reduction in stressors in our set-up might have prevented the emergence of strong age differences in attention or impulsive action. Nonetheless, the absence of a clear age difference in behavioral performance does not exclude the possibility that performance relies on different brain activation patterns during adolescence^44^. The CombiCage SP-5C protocol can provide a unique method for longitudinal studies on the neurocircuitry underlying attention and impulsivity from adolescence to adulthood.

The dose dependent decrease in accuracy after scopolamine administration demonstrates the validity of the protocol to measure attention. The cholinergic system is considered an important mediator of attentional processes^45^,^46^ and multiple studies have reported an effect of scopolamine on accuracy in mice^8^,^23^,^24^,^25^. It has been suggested that scopolamine at doses of 0.1 mg/kg and 1 mg/kg alters motivation and motor functions, clouding the effect on attentional processes^47^. However, the absence of effects on latency measures or number of trials started in this study, excluded an effect of these behavioral constructs on performance. Moreover, using the SP-5C, a full dose-range study, including wash-out days, was completed within two weeks in, exemplifying the potential of this protocol in the context of drug development projects.

The strain differences between C57BL/6J, DBA/2J, BXD16 and BXD62 mice in the SP-5C were in line with previous studies with respect to all performance measures except omissions^20^,^21^. Our data once more confirms that BXD16 mice have poor inhibitory control and attention^48^,^49^. However, the magnitude of strain differences depended on the extent of overtraining, i.e. whether performance values were taken from trials at the beginning or end of the SD1 stage, suggesting strain differences in the stabilization of performance.

DBA/2 J and C57BL/6J are among the most commonly compared mouse strains in behavioral neuroscience. In the SP-5C, DBA/2 J tended to show slightly lower levels of accuracy than C57BL/6J mice, but the difference was not significant. DBA mice were more impulsive in the SP-5C, in line with previous observations in the conventional 5-CSRTT^19^. Others observed significantly lower accuracy in DBA mice, in addition to a difference in impulsivity^26^,^50^. In the SP-5C, DBA/2 J mice seemed less motivated to work for food compared to C57BL/6J mice, as indicated by longer magazine latencies, fewer rewards earned per day and larger weight loss compared to the other strains. These differences were not due to a difference in learning abilities in the SP-5C, as DBA mice were not different from the other strains in the number of trials to criterion in stages SD16 to SD1.5. A similar difference in magazine latency has been reported previously^51^, as well as a reduction in food earned by DBA mice during operant responding^52^ or food consumed under restricted food access^53^. However, DBA mice under food restriction regimes in touchscreen tasks showed shorter reward retrieval latencies^54^,^55^ and DBA mice worked harder and consumed more food when they were housed in an operant chamber for 23 h a day^56^. Although the exact source of the conflict between these results remains unclear, an interaction between dopamine signaling and feeding regime or handling-induced stress sensitivity^57^,^58^ in different set-ups might provide an explanation. Our novel SP-5C protocol combined with the assessment of the neurochemistry underlying performance in C57BL/6J and DBA/2 J mice can provide useful information on this interaction.

With the SP-5C protocol it is now possible to measure impulsivity and attention during short time frames, e.g. that of adolescence, to study the vulnerability to develop, or treatment of, psychiatric disorders. The developed protocol is easy to implement in any set-up in which an operant chamber is connected to a home-cage, be it for mice or rats. Besides the 5-CSRTT, it is quite conceivable that other operant protocols, measuring different cognitive constructs, can be amended to a continuously running 24-h/day protocol for the CombiCage. The data presented here shows that letting animals self-pace their task progression from a home-cage can reduce labor and stressor and can increase the efficiency of testing mouse models of psychiatric disorders as well as novel drug therapies.

## Materials and Methods

### Mice

Male C57BL/6J mice that were tested in the CombiCage at five (n = 8) and ten (n = 9) weeks of age were bred in-house. They were offspring of C57BL/6J mice originally obtained from Charles River Laboratories (L’Arbresle, France) that were bred in-house for a maximum of 3 generations. One week prior to the experiments, mice were single housed on sawdust in standard Makrolon type II cages enriched with cardboard nesting material (7:00/19:00 lights on/off; abrupt phase transition), with water and food *ad libitum* (2018 Teklad, Harlan Laboratories, Horst, The Netherlands).

Conventionally tested male C57BL/6J mice (n = 28) were originally obtained from the Jackson Laboratory and bred by the Neuro-BSIK Consortium for a maximum of 5 generations before testing in 2008^20^,^21^. Around 7-week- old mice were singly housed on sawdust in standard Makrolon type II cages enriched with cardboard nesting material with food (2018 Teklad) and water *ad libitum*. After 1week of habituation body weights were recorded. Over the subsequent 6 days, food was limited to gradually decrease body weight to 90–95% of their initial body weight, before daily training in operant cages commenced.

Male C57BL/6J (n = 21), DBA/2 J (n = 10), BXD16/TyJ (n = 6) and BXD62/RwwJ (n = 6) mice were obtained from The Jackson Laboratory (Bar Harbor, ME, USA) in two separate batches at ~5–12 weeks of age and were allowed to habituate to their novel environment for two weeks before any testing commenced. These animals were first tested in the CognitionWall DL/RL task^34^ before they were tested in the SP-5C task between 10 and 22 weeks of age. Mice from different strains and age were counterbalanced over multiple batches of mice that were tested in a serial manner in the 18 CombiCages that were available.

For the scopolamine experiment, 13-week old male C57BL/6J (n = 18) mice were bred in-house. They were offspring of C57BL/6J mice originally obtained from Charles River Laboratories (L’Arbresle, France) that were bred in-house for a maximum of 3 generations.

All experiments were carried out in accordance with the European Communities Council Directive of 24 November 1986 (86/609/EEC), and with approval of the Animal Experiments Committee of the VU University.

### The CombiCage and self-paced five-choice serial reaction time task protocol

The CombiCage consisted of a home-cage (30 × 30 cm PhenoTyper box, Noldus Information Technology, Wageningen, The Netherlands) connected through a custom made connecting tube to an operant chamber equipped with five stimulus response holes and a food magazine (Fig. 1; MEDNPW-5M, MedAssociates, St Albans, VT, USA). Mice were housed in the CombiCage for the full duration of the experiment. Eighteen CombiCages were placed in a room where the light on/off cycle followed the breeding stable (7:00/19:00 lights on/off; abrupt phase transition). Mice were housed in the home-cage with water and food available *ad libitum* one day before the home-cage was connected to the operant chamber with the tube and the task protocol started at 5 pm. The tube was inserted at the position of the regular food grid in the home-cage and therefore this grid was removed when the task started. The operant chambers of the CombiCages were previously used for the conventional 5-CSRTT experiment, where mice were not continuously housed in their test environment but were tested for 5 days per week, 30 minutes per session.

For the full duration of the protocol mice had to perform the task in order to obtain food reward pellets (Dustless Precision Pellets, 14 mg, Bio-Serve, Frenchtown, NJ, USA). Mice could do so for 24 h a day. The nutritional content of these pellets is similar to regular synthetic lab chow, with some of the starch content replaced by sucrose. Water remained available *ad libitum*. If mice earned fewer than 100 pellets during a day, they would receive the remainder up to 100 for free at 10am. Mice were weighed before and after the experiment, and when they received free reward pellets for 2 or more days in a row. If they earned fewer than 100 pellets for 2 or more days in a row, 0.5–1 g extra chow was given to prevent weight loss beyond 85%.

The SP-5C for the CombiCage was based on the protocol used in the conventional 5-CSRTT setup^18^, the main difference between the two protocols being automatic, performance based, transition between stages in the CombiCage protocol. The SP-5C protocol started off with magazine training, during which reward pellets were dropped at a random fixed inter-trial interval (ITI; 4, 8, 16 and 32 sec), which coincided with switching on the magazine stimulus light. An ITI was only initiated when the previous pellet had been collected, as registered by a magazine response, after which the magazine stimulus light was switched off. This training stage ended when the mouse had received 50 rewards. The next training stage (T1) commenced immediately thereafter, during which a trial started with the illumination of all five stimulus response holes. A response into any of these holes switched off the light in all five holes, switched on the stimulus light in the magazine and delivered a food reward into the magazine. A new trial was initiated when the mouse collected the reward. This training stage ended when the mouse had received 50 rewards after which training 2 (T2) commenced. In this stage, a trial was started by a response in the illuminated food magazine which caused the magazine light to switch off. After a delay of 5 seconds (ITI) one stimulus light switched on. Responses into the non-illuminated holes were without consequence. A response in the illuminated hole switched off the stimulus light, switched on the magazine light and dropped a reward into the magazine. The collection of this reward switched off the magazine light for 20 seconds, after which the magazine light switched on again and a new trial could be initiated. During the 20 seconds consumption interval, responses in the food magazine were without consequences. Stage T2 lasted until a mouse had earned 100 rewards.

Hereafter, the actual 5-CSRTT procedure started. In this part of the protocol, similarly to T2, a trial was started by a nose poke in the illuminated magazine, which switched off the magazine light. After a 5 sec ITI, a stimulus was switched on in one of the five stimulus response holes for a limited duration (stimulus duration; SD). A response in the correct stimulus hole, during stimulus presentation or within the limited hold of 4 seconds after termination of the stimulus, switched on the magazine light and delivered a food reward. The collection of this reward switched off the magazine light for 20 seconds, after which the magazine light switched on again and a new trial could be initiated. An incorrect response into a non-illuminated stimulus hole, a premature response during the 5 sec ITI, or an omission of a response, resulted in a 5-second time-out period, during which all stimulus lights were switched off. When the time-out period ended, the magazine light was switched on and the mouse could start the next trial.

In the first 5-CSRTT stage, the stimulus duration was set to 16 seconds and this duration was gradually decreased in subsequent stages to 8, 4, 2, 1.5 and 1 sec if the mouse reached the criterion (started trials >50, accuracy >60% and [omissions <30% or number of correct responses > = 200]). Percentage accuracy and percentage omission were calculated online using a moving average with window size 20. The performance criteria for stage transition in the conventional protocol were: omissions <30%, accuracy >60%, started trials >50 or after 10 sessions.

Five and ten week old C57BL/6J mice were tested on a protocol where they had to complete five SD1 stages by reaching the criterion described above, before they received a variable ITI (vITI) stage of 500 trials (fixed random ITI of 5, 7.5 and 12.5 s), followed by 500 trials in SD1, followed by a variable SD (vSD) stage of 500 trials (fixed random SD of 1, 0.5 and 0.2 s), after which the protocol was ended. Animals in the BXD experiment were given 1000 trials in SD1 before the protocol was ended. In the conventional 5-CSRTT protocol, the mice were trained for 10 sessions in SD1^20^.

### Drug administration

Scopolamine hydrobromide (Scopolamine hereafter), a muscarinic acetylcholine receptor antagonist, was purchased from Sigma-Aldrich (Zwijndrecht, The Netherlands), and dissolved in 0.9% saline. Eighteen C57BL/6J mice were started in the above described SP-5C protocol on Tuesday. On the next Monday morning all mice had reached the SD1 stage. Scopolamine (0.3 and 1 mg/kg) and saline were administered at 10 ml/kg intraperitoneally (i.p.) according to a Latin square design on Monday, Wednesday and Friday between 6.30 and 6.40 pm, while the 5-CSRTT protocol kept running continuously in the SD1 stage until it was manually stopped on Saturday morning. Because scopolamine’s half-life is 40 min^59^, data generated from 7–9 pm on Sunday till Friday was extracted and analyzed for performance on drug and no-drug days.

### Data analysis

Accuracy, a measure of sustained divided attention^6^, was defined as [100 × (number of correct responses)/(number of correct and incorrect responses)]. The percentage of premature responses, a measure of impulsivity^6^, was defined as [100 × (number of premature responses)/(number of premature + number of correct and incorrect responses)]. The percentage of omissions, a measure of either motivation or attention, was defined as [100 × (omissions)/(omissions + number of correct and incorrect responses)]. Correct response latency represents the time between the onset of the stimulus and a nose-poke in the correct hole and when not correlated to magazine latency it presumably represents the speed of decisional processes. Response variability, a measure of lapses in attention^19^,^20^,^27^,^28^, was defined by the standard deviation of correct response latencies within a mouse. Magazine latency, considered to gage motivation, was defined as the time between a correct response and a poke into the food magazine. The complete profile of all latency measures can provide information about motor function in the animals^6^. Trials with a magazine latency >10 sec were excluded from further analysis.

Baseline performance in the conventional 5-CSRTT previously reported^18^,^20^ was calculated based on session 6 until 10 in SD1. To determine trends in performance in the BXD experiment in the CombiCage, the 1000 SD1 trials these mice received were split into five bins of 200 trials for data analysis purposes.

The effect of protocol and age was tested using a linear model in which planned contrasts were set comparing protocol and age. For the vITI and vSD stages, the duration of the ITI or the SD was included as a within subject factor into a multilevel linear model. Performance in the BXD strains was analyzed using a multilevel linear model, followed by Tukey post-hoc test comparing strains and bins of SD1 trials. The effect of scopolamine was analyzed using a factorial ANOVA with drug dose and day of administration as factors. When the main effect of the drug was significant, Dunnett post-hoc tests were performed to compare each scopolamine dose to saline. Correlation analyses were performed using the non-parametric Kendall tau correlation coefficient using data of individual mice, not strain means.

If data was not normally distributed, this was corrected by either arcsine transformation, when it concerned percentage data, or log transformation of the data. All data analyses were performed in R. Error bars in graphs represent the standard error of the mean.

## Additional Information

**How to cite this article**: Remmelink, E. *et al*. A one-week 5-choice serial reaction time task to measure impulsivity and attention in adult and adolescent mice. *Sci. Rep.* **7**, 42519; doi: 10.1038/srep42519 (2017).

**Publisher's note:** Springer Nature remains neutral with regard to jurisdictional claims in published maps and institutional affiliations.

## Figures and Tables

**Table 1:** Overview of experimental details of adolescent and adult C57BL/6J mice tested in the self-paced 5-CSRTT (SP) and adult C57BL/6J mice previously tested in the conventional 5-CSRTT (Conv).

	C57BL/6J 5w SP	C57BL/6J 10w SP	Adult C57BL/6J Conv
Number of mice	8	9	28
Age in weeks at start	5.0	10.1	~8
Days to finish until SD1	3.7 ± 0.5	4.8 ± 1	51.5 ± 13
Started trials all SD stages incl. SD1	1147 ± 53	1560 ± 109	2000 ± 605
Started trials vITI	500	500	59 ± 0
Started trials vSD	500	500	52 ± 2
% of SD trials during the dark phase	82 ± 7	83 ± 7	N/A
Rewards/day	184 ± 15	179 ± 12	N/A
Food/day grams	2.6 ± 0.2	2.5 ± 0.2	N/A
Free rewards @ 10 am (mean/day)	0.6 ± 1.1	0.8 ± 1.2	N/A
Number of mice receiving additional food on more than 2 days	0	0	N/A
Weight difference %	1.3 ± 4.1	−7.4 ± 2.9	N/A

Values represent mean ± sd.

**Table 2:** Overview of experimental details of BXD mice tested in the SP-5C.

	BXD16/TyJ	BXD62/RwwJ	C57BL/6J	DBA/2 J
Number of mice	6	6	21	10
Age in weeks at start	10.6 ± 0.7	14.9 ± 0.5	16.3 ± 3.6	17.8 ± 2.2
Days to finish until SD1	5.7 ± 0.8	6.3 ± 1.3	5.2 ± 0.9	7.2 ± 2.8
Started trials all SD stages incl. SD1	2064 ± 163	1700 ± 199	1612 ± 37	1906 ± 108
% of 1000 SD1 trials started during the dark phase	91 ± 5	91 ± 4	91 ± 3	59 ± 19
Rewards/day	155 ± 28	146 ± 22	164 ± 18	127 ± 43
Food/day grams	2.2 ± 0.4	2.0 ± 0.3	2.3 ± 0.3	1.8 ± 0.6
Free rewards @ 10am (mean/day)	4.8 ± 6.8	13.6 ± 10.5	1.4 ± 3.7	17.2 ± 20.7
Number of mice receiving additional food on more than 2 days	0	0	0	3
Weight difference %	0.8 ± 5.3	3.0 ± 2.6	−2.5 ± 2.1	−13.1 ± 4.7

Values represent mean ± sd.
